# Quorum Sensing: Not Just a Bridge Between Bacteria

**DOI:** 10.1002/mbo3.70016

**Published:** 2025-03-30

**Authors:** Derun Liu, Yonglin Lu, Ziyun Li, Xin Pang, Xueyan Gao

**Affiliations:** ^1^ Medical Science and Technology Innovation Center Shandong First Medical University & Shandong Academy of Medical Sciences jinan China; ^2^ State Key Laboratory of Microbial Technology Shandong University Qingdao China

**Keywords:** bacterial communication, interaction, mechanisms, quorum sensing

## Abstract

The study of quorum sensing (QS) has gained critical importance, offering insights into bacterial and microorganism communication. QS, regulated by autoinducers, synchronizes collective bacterial behaviors across diverse chemical signals and target genes. This review highlights innovative approaches to regulating QS, emphasizing the potential of quorum quenching and QS inhibitors to mitigate bacterial pathogenicity. These strategies have shown promise in aquaculture and plant resistance, disrupting QS pathways to combat infections. QS also provides opportunities for developing biosensors for early disease detection and preventing biofilm formation, which is critical to overcoming antimicrobial resistance. The applications of QS extend to cancer therapy, with targeted drug delivery systems utilizing QS mechanisms. Advancements in QS regulation, such as the use of nanomaterials, hydrogels, and microplastics, provide novel methods to modulate QS systems. This review explores the latest developments in QS, recognizing its significance in controlling bacterial behavior and its broad impacts on human health and disease management. Integrating these insights into therapeutic strategies and diagnostics represents a pivotal opportunity for medical progress.

## Graphical abstract

Quorum quenching and quorum sensing inhibitors show significant potential in regulating bacterial quorum sensing systems and have been widely applied across various fields, including cancer treatment, antimicrobial resistance, marine management, microplastic reduction, hydrogel technology, and nanomaterials development.

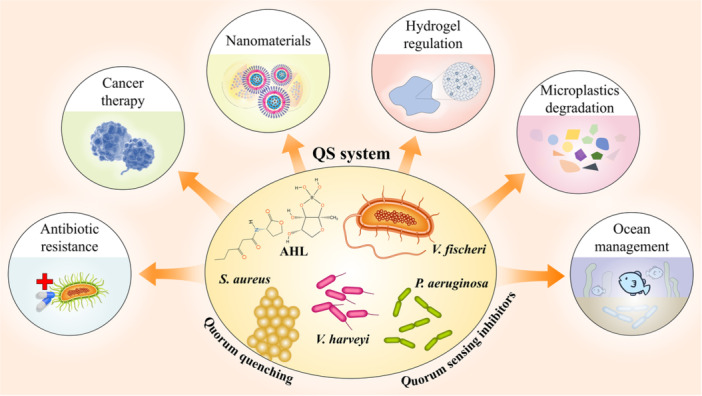

## 1 Introduction

Quorum sensing (QS) is a complex communication system among bacteria. QS enables bacterial groups to synchronously coordinate their behavior in response to fluctuations in population density and species composition in neighboring communities. The production, detection, and response to extracellular signaling molecules known as autoinducers are central to this process (Waters and Bassler 2005). As the bacterial population increases, the concentration of autoinducer molecules rises. Once a critical concentration threshold is attained, the bacteria can detect autoinducer molecules and initiate a specific response. QS is essential for various finely adapted behaviors, such as bacterial virulence (Winson et al. 1995), biofilm formation (Cvitkovitch et al. 2003; Solano et al. 2014; L. Xu et al. 2006), drug resistance (Pumbwe et al. 2008), bioluminescence (Engebrecht et al. 1983; Engebrecht and Silverman 1984, 1987), and response to environmental changes (Lee and Zhang 2015). With the advent of QS systems, bacteria are no longer regarded as single‐celled living organisms that can only carry out simple processes. Most processes regulated by QS are ineffective when conducted by a single bacterium acting in isolation. Thus, QS enables bacteria to cooperate among individual cells, thereby achieving high levels of coordination. There are differences between QS systems due to adaptations for specific species in particular living conditions. In diverse bacterial species, autoinducers exhibit high specificity, with the type of signal, outputs, transduction mechanisms, and receptors all reflecting the distinct biology of each microorganism (Whiteley et al. 2018).

In the 1960s and 1970s, the first reports of QS revealed that extracellular molecules were essential for competence in *Streptococcus pneumoniae* (Tomasz 1965) and bioluminescence in marine pathogenic bacteria (Nealson et al. 1970). In *Vibrio fischeri*, the production of light is closely related to the cell density (Bassler et al. 1994). Bioluminescence is expressed only when the density of *V. fischeri* cells reaches a sufficiently high level. In the 1980s, the discovery of the genes *luxI* and *luxR*, which encode bioluminescence and the autoinducer 3‐oxo‐C_6_‐HSL used in this system, respectively, provided a complete picture of the QS system (Eberhard et al. 1981; Engebrecht et al. 1983). By the 1990s, DNA sequencing unveiled LuxI and LuxR homologs in various bacteria, leading to the concept of QS (Fuqua et al. 1994). This communication system, which is present in both Gram‐negative and Gram‐positive bacteria, was initially studied in pathogenic microbes, after which the research focus shifted to the broader implications of bacterial communication. Further research uncovered the autoinducer‐2 (AI‐2) in *Vibrio harveyi* (X. Chen et al. 2002), expanding QS beyond bacterial species to include multispecies and eukaryotes (Federle and Bassler 2003). Interspecies communication makes it possible to artificially intervene in bacterial communication within the human body. Later reports demonstrated that in many animal and plant pathogens, the virulence of QS mutants was greatly reduced (Balaban and Novick 1995). Through employing chemical intervention to inhibit the communication between bacteria (Deng et al. 2020) or between bacteria and viruses (Silpe et al. 2023) in the human body, it is possible to use QS to treat diseases.

## 2 Gram‐Negative Bacteria

Engebrecht et al.'s research on *V. fischeri* laid the foundation for studying QS in Gram‐negative bacteria (Engebrecht et al. 1983; Engebrecht and Silverman 1984, 1987) and identified the LuxI–LuxR system and acyl‐homoserine lactone (AHL) signaling molecules. The composition of the acyl chain varies from 4 to 16 carbon atoms (Table 1). The signaling molecules exhibit a high degree of specificity due to their structural features (Watson et al. 2002). The most prevalent signaling molecule found in Gram‐negative bacteria is the *N*‐acyl‐homoserine lactone derivative (Whitehead et al. 2001). Table 2 lists the diverse and complex QS communication networks of Gram‐negative bacteria.

**Table 1:** List the abbreviations and structures of common autoinducers.

Signaling molecules	Abbreviations	Common bacteria	Structures
*N*‐butanoyl‐l‐homoserine lactone	C_4_‐HSL	*Aeromonas, Serratia, Pseudomonas aeruginosa*	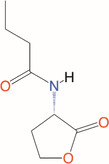
*N*‐hexanoyl‐l‐homoserine lactone	C_6_‐HSL	*Aeromonas, Erwinia, Serratia, Yersinia, Pseudomonas aureofaciens*	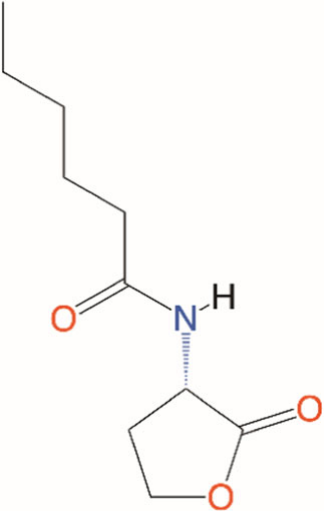
*N*‐Heptanoyl‐l‐homoserine lactone	C_7_‐HSL	*Serratia marcescens*	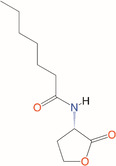
*N*‐octanoyl‐l‐homoserine lactone	C_8_‐HSL	*S. marcescens, Yersinia pseudotuberculosis*	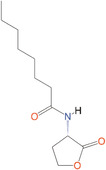
*N*‐decanoyl‐l‐homoserine lactone	C_10_‐HSL	*Aeromonas salmonicida, Erwinia chrysanthemi*	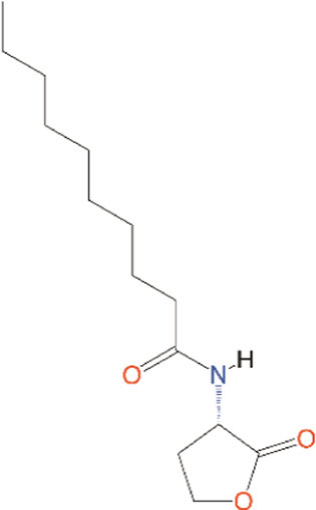
*N*‐(3‐oxohexanoyl)‐l‐homoserine lactone	3‐oxo‐C_6_‐HSL	*A. salmonicida, Erwinia, Serratia, Vibrio fischeri, Yersinia*	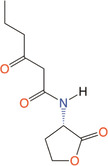
*N*‐(3‐oxooctanoyl)‐l‐homoserine lactone	3‐oxo‐C_8_‐HSL	*Agrobacterium tumefaciens, Y. pseudotuberculosis*	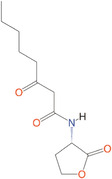
*N*‐(3‐oxodecanoyl)‐l‐homoserine lactone	3‐oxo‐C_10_‐HSL	*Vibrio anguillarum, Y. pseudotuberculosis*	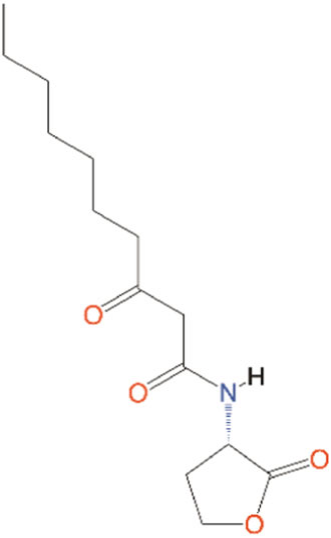
*N*‐(3‐oxododecanoyl)‐l‐homoserine lactone	3‐oxo‐C_12_‐HSL	*P. aeruginosa*	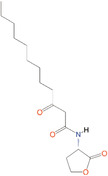
*N*‐(3‐hydroxy‐7‐*cis*‐tetradecenoyl)‐l‐homoserine lactone	3OH‐C_14:1_‐HSL	*Rhizobium leguminosarum*	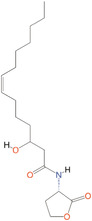
*N*‐(3‐oxo‐*cis*‐11‐hexadecenoyl) homoserine lactone	3‐oxo‐C_16:1_‐HSL	*Rhizobium meliloti*	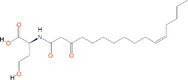
2‐Heptyl‐3‐hydroxy‐4‐quinolone	PQS	*Pseudomonas*	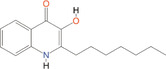
*N*‐(3‐hydroxybutanoyl)‐l‐homoserine lactone	HAI‐1	*Vibrio harveyi*	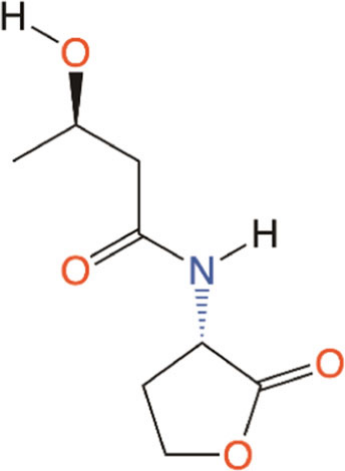
(*S*)‐3‐hydroxytridecan‐4‐one	CAI‐1	*V. harveyi, Vibrio cholerae*	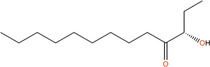
3A‐methyl‐5,6‐dihydro‐furo (2,3‐d) (1,3,2) dioxaborole‐2,2,6,6A‐tetraol	AI‐2	*V. harveyi*	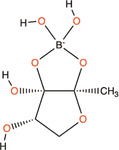

Abbreviations: AHL, acyl‐homoserine lactone; AI‐2, autoinducer‐2; HSL, homoserine lactone; PQS, *Pseudomonas* quinolone signal.

**Table 2:** List the autoinducers and their main roles in the LuxI/R family QS system of identified Gram‐negative bacteria.

Bacteria	LuxI/R Homologs	Abbreviations	Genes involved	Phenotype	Reference
*Aeromonas hydrophila*	AhyI/AhyR	C_4_‐HSL, C_6_‐HSL	*litR*	Production of serine protease	B. Sun, Luo, et al. (2021)
*Aeromonas salmonicida*	AsaI/AsaR	C_4_‐HSL, C_6_‐HSL, C_10_‐HSL, 3‐oxo‐C_6_‐HSL	*aspA*	Production of serine protease	Schwenteit et al. (2011)
*Agrobacterium tumefaciens*	TraI/TraR	3‐oxo‐C_8_‐HSL	*tra, trb*	Ti plasmid conjugal transfer	Lang and Faure (2014)
*Burkholderia cepacia*	CepI/CepR	C_8_‐HSL	Unknown	Protease, siderophore, and biofilm production	Lewenza et al. (1999)
*Chromobacterium violaceum*	CviI/CviR	C_6_‐HSL	Unknown	Chitinolytic activity regulation	Chernin et al. (1998)
*Erwinia carotovora*	a. ExpI/ExpR b. CarI/CarR	3‐oxo‐C_6_‐HSL	*Car* operon	a. Production of exoenzymes b. Production of carbapenem	Vieira et al. (2020)
*Erwinia chrysanthemi*	ExpI/ExpR	a. 3‐oxo‐C_6_‐HSL b. C_6_‐HSL c. C_10_‐HSL	*pel, pecS‐pecM, pecT*	Regulation of pectinase production	Nasser et al. (1998)
*Erwinia stewartii*	EsaI/EsaR	3‐oxo‐C_6_‐HSL	*cps* gene system	Synthesis of capsular polysaccharides	Koutsoudis et al. (2006)
*Pseudomonas aeruginosa*	a. LasI/LasR b. RhlI/RhlR	a. 3‐oxo‐C_12_‐HSL b. C_4_‐HSL	a. *lasA, lasB, toxA* b. *rhlAB, lasB, rpoS*	a. Virulence factor production and biofilm formation b. Encoding rhamnosyltransferase and the expression of stationary‐phase sigma factor	Latifi et al. (1996), O'Loughlin et al. (2013), and Ochsner et al. (1994)
*Pseudomonas aureofaciens*	PhzR/PhzI	C_6_‐HSL	*phzXYFABCD*	Regulation of phenazine production	Maddula et al. (2006)
*Pseudomonas fluorescens* 2P24	Pcol/PcoR	a. C_6_‐HSL b. 3‐oxo‐C_8_‐HSL	*gac, rsm, phlACBD*	Rhizosphere colonization	X. Yu et al. (2022)
*Rhizobium etli*	RaiI/RaiR	Unknown	Unknown	Nitrogen‐fixing nodules formation	Rosemeyer et al. (1998)
*Rhizobium leguminosarum*	a. RhiI/RhiR b. CinI/CinR c. TraI/TraR d. RaiI/RaiR	a. C_6_‐HSL b. 3OH‐C_14:1_‐HSL c. short chain AHLs d. C_6_‐HSL	a. *rhiABC* b. *rhiI, traI* c. *trb*	Inhibition of nodulation, production of small bacteriocin	Wisniewski‐Dyé and Downie (2002)
*Rhizobium meliloti*	SinI/SinR	3‐oxo‐C_16:1_‐HSL	*expR*	Galactoglucan biosynthesis	Calatrava‐Morales et al. (2018)
*Serratia liquefaciens*	SwrI/SwrR	C_4_‐HSL, C_6_‐HSL	*swrA*	Regulating cluster activity, formation of unique biofilm structures	Remuzgo‐Martínez et al. (2015)
*Serratia marcescens*	a. SwrI/SwrR b. SpnI/SpnR c. SmaI/SmaR	a. C_4_‐HSL, C_6_‐HSL b. C_6_‐HSL, 3‐oxo‐C_6_‐HSL	a. flhDC operon b. lux box	a. Swarming motility and biofilm formation b. Sliding motility, production of biosurfactant, prodigiosin and nuclease c. Swarming motility and biofilm formation	Van Houdt et al. (2007)
		C_7_‐HSL, C_8_‐HSL			
		c. C_4_‐HSL, C_6_‐HSL			
*Sinorhizobium meliloti*	SinI/SinR	long‐chain AHLs	*expR, sinRI*	Production of succinoglycan and galactoglucan (EPS II)	Gurich and González (2009)
*Vibrio anguillarum*	VanI/VanR	3‐oxo‐C_10_‐HSL	Unknown	Unknown	Mauritzen et al. (2023)
*Vibrio fischeri*	LuxI/LuxR	3‐oxo‐C_6_‐HSL	*luxCDABE*	Bioluminescence	Graf and Ruby (1998)
*Yersinia enterocolitica*	YenI/YenR	C_6_‐HSL, 3‐oxo‐C_6_‐HSL	*spyA*	Swimming motility, cell attachment and virulence plasmid maintenance	Ng et al. (2018)
*Yersinia pestis*	YspI/YspR	3‐oxo‐C_6_‐HSL, C_6_‐HSL	*crp*	Virulence regulation	Ritzert et al. (2019)
*Yersinia pseudotuberculosis*	a. YpsI/YpsR b. YtbI/YtbR	3‐oxo‐C_6_‐HSL, 3‐oxo‐C_8_‐HSL, 3‐oxo‐C_10_‐HSL, C_6_‐HSL, C_8_‐HSL	*nagC*	Motility and cellular aggregation	Wiechmann et al. (2023)

Abbreviations: AHL, acyl‐homoserine lactone; HSL, homoserine lactone.

**Figure 1:**
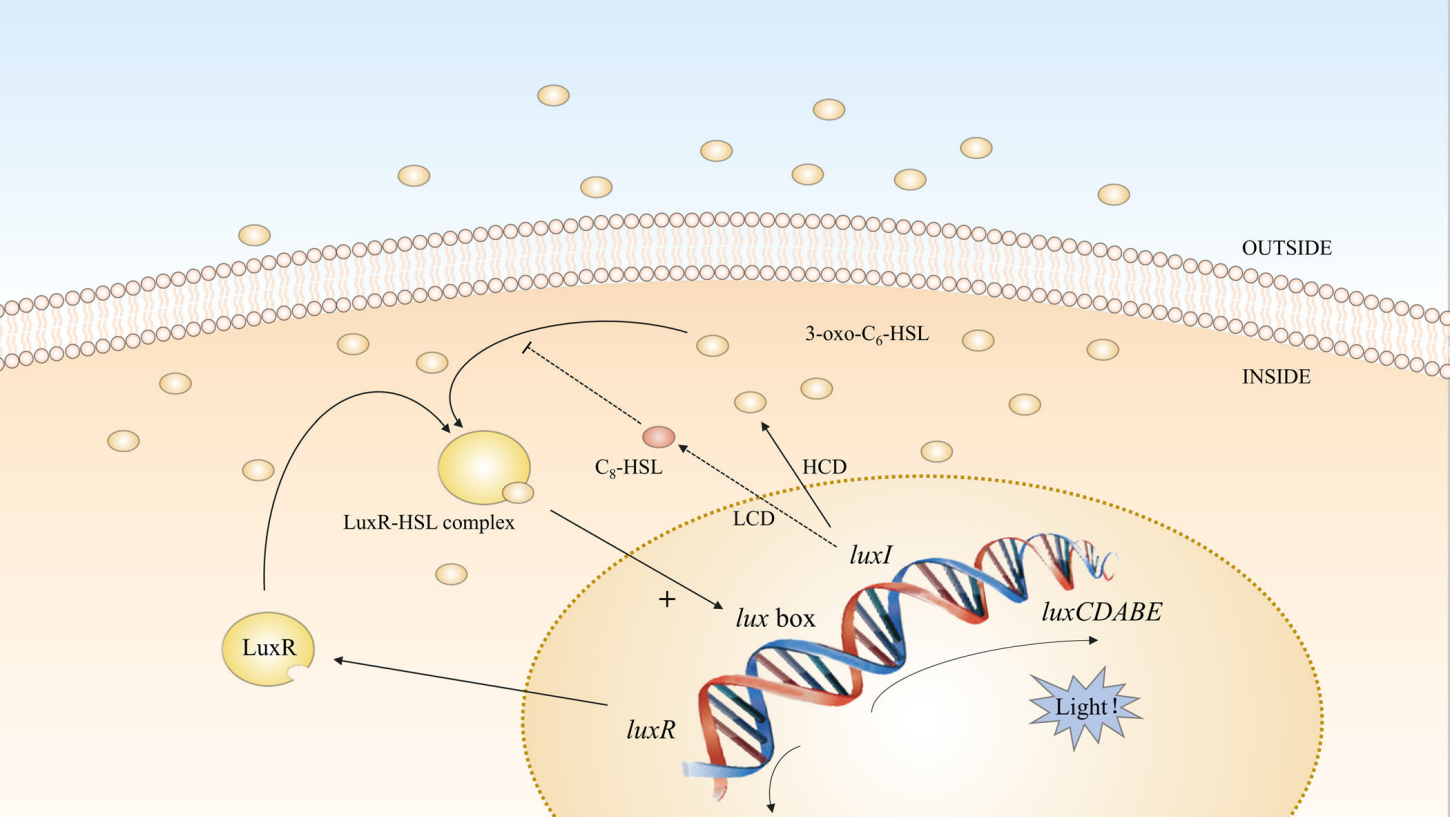
Regulation of bioluminescence in *V. fischeri*. At LCD, *luxI* synthesis of C_8_‐HSL would compete to inhibit the binding of 3‐oxo‐C_6_‐HSL to LuxR. When the cell density reaches a threshold, it is able to produce enough 3‐oxo‐C_6_‐HSL to combine with LuxR to form a complex, thereby initiating the transcription of *luxCDABE* and forming bioluminescence. Small ellipses represent signaling molecules and large ellipses represent proteins. HCD, high cell density; HSL, homoserine lactone; LCD, low cell density.

*V. fischeri* produces light within the light organ of squid as the population density reaches a critical threshold. This light helps the cephalopod avoid predation at night, which benefits the bacteria in turn (Graf and Ruby 1998). LuxI produces the autoinducer 3‐oxo‐C_6_‐HSL (Table 1), which activates LuxR to induce *luxCDABE* transcription (Engebrecht et al. 1983; Engebrecht and Silverman 1984). This leads to luciferase synthesis and bioluminescence. A positive feedback loop maintains bioluminescence via increasing acyl‐HSL levels (Moré et al. 1996). At low cell densities (LCDs), LuxI synthesizes C_8_‐HSL (Figure 1), delaying autoinduction to conserve energy (Callahan and Dunlap 2000; Eberhard 1972; Kuo et al. 1996; Nealson et al. 1970). The QS system of many Gram‐negative bacteria is highly specific, although homologous to the LuxIR type. A particular AHL molecule is only detected by the species that produced it (Watson et al. 2002).

**Figure 2:**
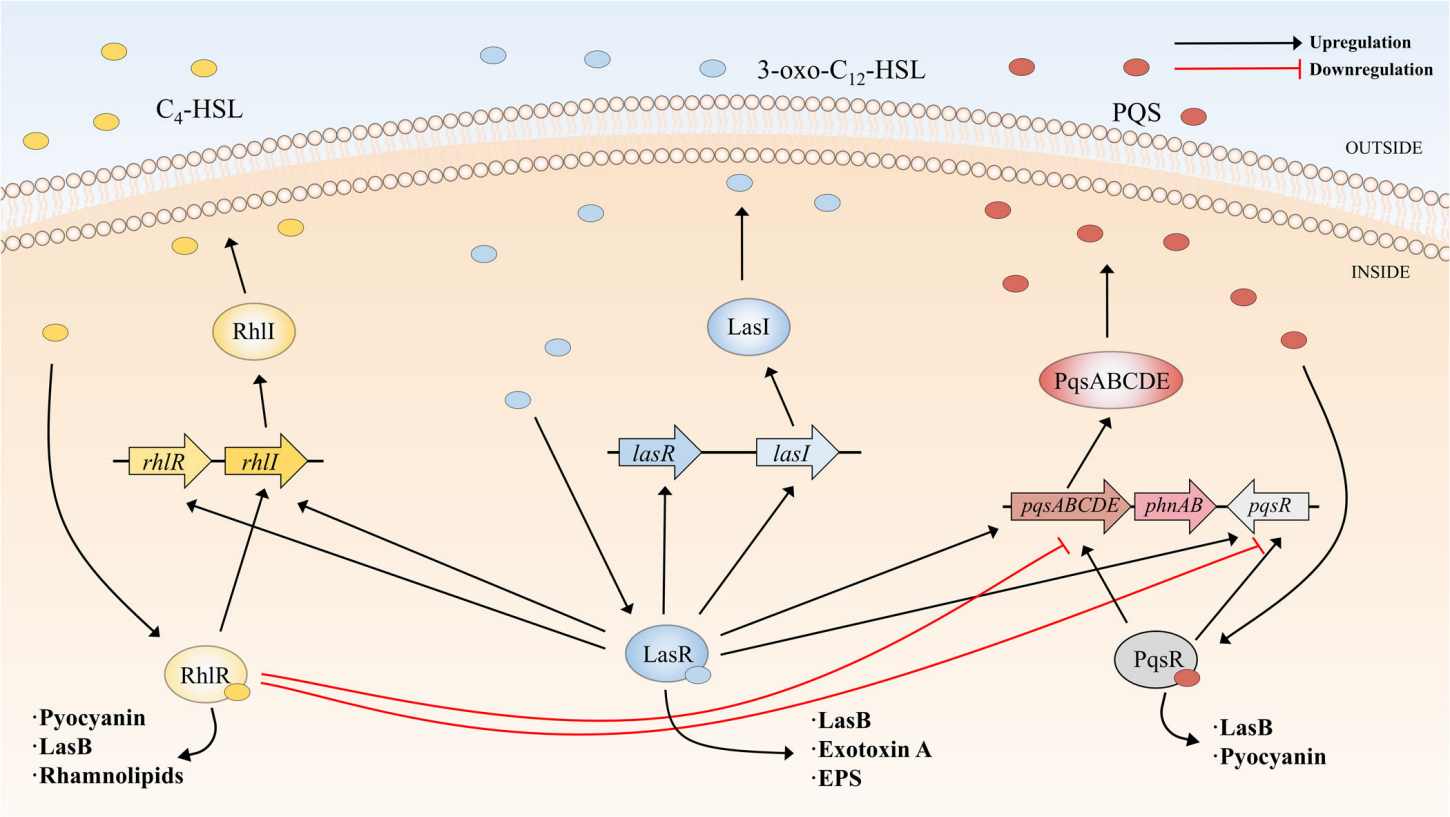
QS regulatory system in *Pseudomonas aeruginosa*. The yellow section represents the Rhl system. The blue part represents the Las system. The red part represents the Pqs system. The black arrow indicates upregulation and the red blocking arrow indicates downregulation. The small ellipses represent signaling molecules. The large ovals represent proteins. The Las system activates the Rhl and Pqs systems. However, the Rhl system has an inhibitory effect on the Pqs system. The three systems interact to express the production of various virulence factors. HSL, homoserine lactone; PQS, Pseudomonas quinolone signal; QS, quorum sensing.

*Pseudomonas aeruginosa* primarily infects immunocompromised individuals, such as patients with AIDS, cystic fibrosis (CF), and urinary tract infections (Azam and Khan 2019). The pathogenicity of *P. aeruginosa* is linked to virulence factors regulated by a unique QS system, which involves two sets of AHL‐mediated signaling circuits and a pathway mediated by 2‐alkyl‐4‐quinolones (Latifi et al. 1996; Pesci et al. 1999) (Figure 2). LasI in *P. aeruginosa* produces 3‐oxo‐C_12_‐HSL (Table 1), which binds to LasR, triggering a feedback loop that enhances the expression of LasI (Gambello and Iglewski 1991; Passador et al. 1993; Pearson et al. 1994; Schuster and Peter Greenberg 2006; Venturi 2006). This means that concentration of 3‐oxo‐C_12_‐HSL is closely associated with *P. aeruginosa*'s virulence. The second QS system consists of RhlI and RhlR, with RhlI synthesizing *N*‐butanoyl‐l‐homoserine lactone (C_4_‐HSL) (Table 1) (Pearson et al. 1995; Winson et al. 1995). The RhlR‐HSL complex also forms a positive feedback loop, and these two signaling systems interact in sequence (M. B. Miller and Bassler 2001). The RhlIR system relies on the LasIR system for activation (Latifi et al. 1996; Pesci et al. 1997). Both systems regulate the production of specific virulence factors, including the elastin hydrolyzing protein LasB (Gambello and Iglewski 1991; Passador et al. 1993; Pearson et al. 1994; Pearson et al. 1995; Pearson et al. 1997; Winson et al. 1995). In addition, Pesci et al. identified a third signaling molecule, 2‐heptyl‐3‐hydroxy‐4‐quinolone, referred to as the *Pseudomonas* quinolone signal (PQS) (Table 1). This signaling molecule is produced by the *pqsABCDE* operon and binds to PqsR to regulate the expression of traits (Cao et al. 2001; Pesci et al. 1999). The Las and Rhl systems exert positive and negative regulatory effects on the PQS system, respectively (Brouwer et al. 2014; Gallagher et al. 2002). Overall, *P. aeruginosa*'s QS system is more complex than that of *V. fischeri*. The three systems are interconnected and collectively regulate the expression of virulence factors (Figure 2). Rhl and Pqs systems can regulate pyocin detected in CF patients (Gallagher et al. 2002; Mukherjee et al. 2017). All three systems participate in the generation of LasB (Brint and Ohman 1995; Diggle et al. 2003; Pesci et al. 1997). Bacteriocin and LasB play important roles in the pathogenesis of *P. aeruginosa* (S. Hall et al. 2016; Kuang et al. 2011), which also leads to the prevalence of *P. aeruginosa* in hospitals (Weiner et al. 2016). This complexity is a focus of ongoing research, particularly regarding the relationship between QS and drug resistance (Reynolds and Kollef 2021).

**Figure 3:**
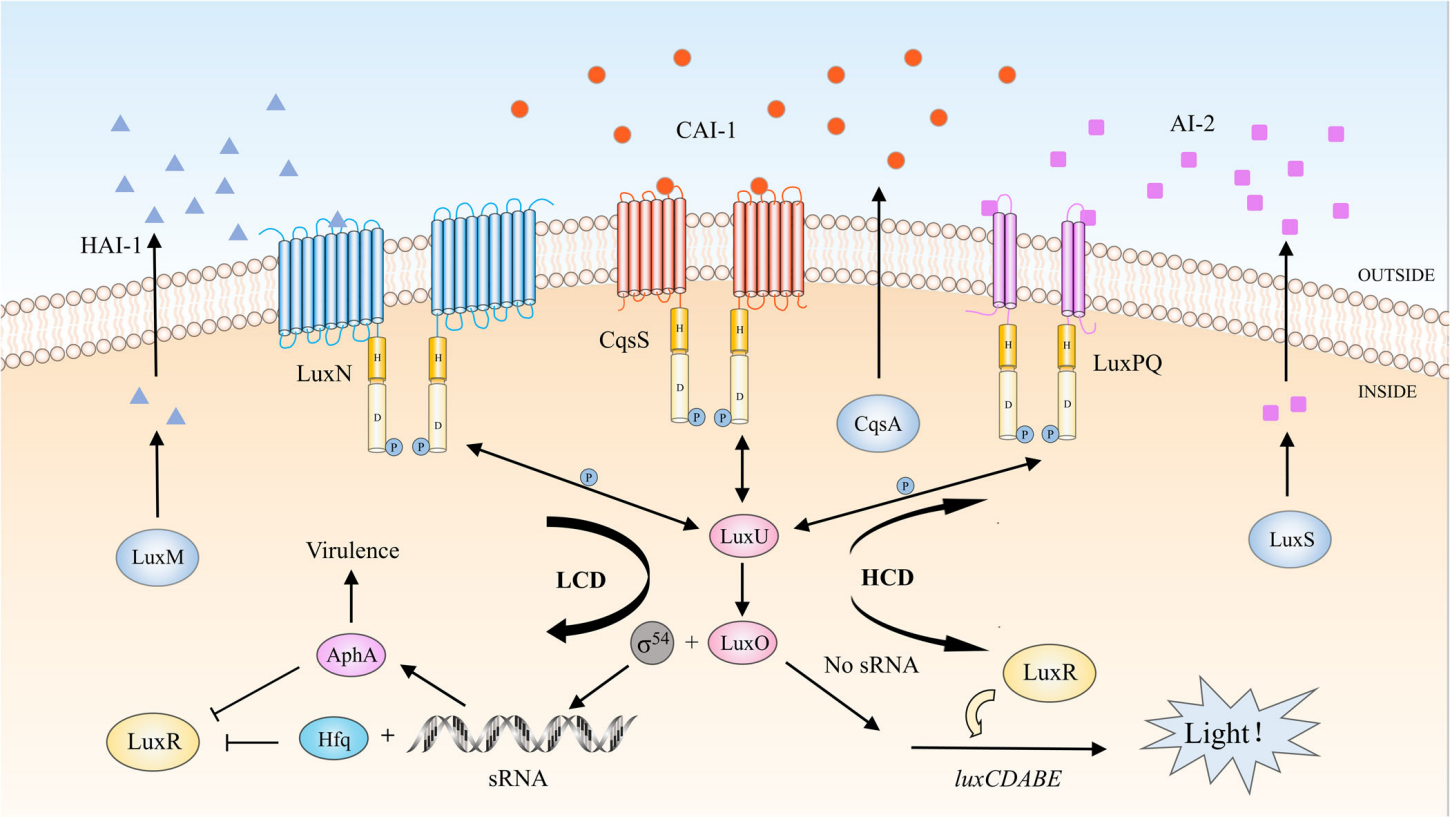
*Vibrio harveyi* reacts to three autoinducers. HAI‐1, AI‐2, and CAI‐1 are replaced by blue triangles, purple rectangles, and red circles, respectively. The large ovals represent proteins. The cylinders H and D denote conserved histidine and conserved aspartate, respectively. At LCD, LuxN, and LuxQ undergo autophosphorylation, sequentially transferring phosphate to LuxU and LuxO. Qrr‐sRNA was activated to express AphA and inhibit LuxR. AphA at high levels is a major regulator at LCD. At HCD, LuxN, and LuxQ switch from kinases to phosphatases. Inactive LuxO is not phosphorylated. Disinhibition of LuxR by sRNA leaves LuxR at a high level. LuxR binds to the *luxCDABE* promoter, activating transcription. So, *V. harveyi* emits light. Double‐headed arrows indicate the direction of transfer of phosphorylation. Phosphorylation shifted toward LuxO at LCD and the opposite at HCD. AI‐2, autoinducer‐2; CAI‐1, (*S*)‐3‐hydroxytridecan‐4‐one; HAI‐1, *N*‐(3‐hydroxybutanoyl)‐l‐homoserine lactone; HCD, high cell density; LCD, low cell density; QS, quorum sensing; sRNA, small RNA.

Bacteria utilize QS circuits with high specificity to distinguish themselves from other species, enabling coordinated behaviors within bacterial communities. In addition to self‐recognition, bacteria possess mechanisms to detect other species. *V. harveyi* possesses three QS systems for inter‐ and intraspecific communication that produce distinct signal molecules (Figure 3). Among them, *N*‐(3‐hydroxybutanoyl)‐l‐homoserine lactone (HAI‐1) facilitates intraspecies communication (Bassler et al. 1993), while (*S*)‐3‐hydroxytridecan‐4‐one (CAI‐1) operates within the *Vibrio* genus, and AI‐2 is detectable by a broad range of bacteria, enabling cross‐species signaling (Table 1) (Bassler et al. 1994). These systems involve Lux proteins in phosphorylation and dephosphorylation cascades that regulate LuxR and AphA (Ball et al. 2017; Freeman and Bassler 1999b; Freeman et al. 2000). AphA and LuxR are the major QS‐regulated transcription factors (Rutherford et al. 2011). Among them, LuxR is only responsible for transcription rather than acting as a signaling receptor (Showalter et al. 1990). At an LCD, sRNAs inhibit LuxR (Lenz et al. 2004; Lilley and Bassler 2000), while at a high cell density (HCD), LuxR expression leads to bioluminescence (Freeman and Bassler 1999a; Lilley and Bassler 2000). This complex QS network underscores the versatility of bacterial communication, benefiting multispecies survival in shared environments (Liu et al. 2022).

Beyond *V. fischeri*, *P. aeruginosa*, and *V. harveyi*, diverse LuxIR‐based QS systems have been described (Table 2). For example, *Agrobacterium tumefaciens* induces crown gall disease through horizontally transferring T‐DNA from virulence tumor‐inducing (Ti) plasmids to plants, activating its QS system (Chevrot et al. 2006; Chilton et al. 1977; Haudecoeur and Faure 2010). *A. tumefaciens*' QS system regulates the spread of Ti plasmid with an *N*‐(3‐oxooctanoyl)‐l‐homoserine lactone signal (H. B. Zhang et al. 2002). *Pseudomonas aureofaciens* produces phenazine antibiotics, giving it a competitive edge against fungi and enabling it to serve as a biocontrol agent (Mazzola et al. 1992). In the QS circuit of *P. aureofaciens*, *N*‐hexanoyl‐l‐homoserine lactone is synthesized by PhzI and binds to PhzR to regulate the expression of target genes (Wood et al. 1997; Wood and Pierson 1996). *Pseudomonas fluorescens* 2P24 utilizes the PcoIR system to regulate colonization around the rhizosphere and protect plant roots from disease (X. Yu et al. 2022). *Sinorhizobium meliloti* regulates the production of succinoglycan and EPS II through the AHL synthetase SinI and the receptor ExpR, allowing it to invade the nodules of *Medicago* legumes to form a symbiotic relationship through nitrogen fixation (Gao et al. 2005).

The variety of AHLs highlights the ubiquity of QS systems in these bacteria. Research has shown that QS in Gram‐negative bacteria involves not only LuxI/R circuits but also two‐component systems, as seen in *Serratia* (Van Houdt et al. 2007). As mentioned above, a wide variety of QS systems in Gram‐negative bacteria are closely associated with virulence factors and pathogenicity. Thus, regulating QS to alter, reduce, or even inhibit the production of bacterial pathogenicity is a promising research direction (Landman et al. 2018).

## 3 Gram‐Positive Bacteria

Gram‐positive bacteria utilize self‐inducing peptides (AIPs) for QS, unlike Gram‐negative bacteria, which rely on homoserine lactones (HSLs). In Gram‐negative bacteria, AHL signaling molecules usually activate responses by diffusing and binding to LuxR homolog receptors. By contrast, the QS system of Gram‐positive bacteria includes a two‐component system with a phosphorylation cascade and Rap, Rgg, NprR, PlcR and PrgX family that forms intracellular complexes. Signal transduction relies on the switch between the phosphorylation and dephosphorylation of sensor proteins to guide cascade reactions. (Neiditch et al. 2017). AIPs require exporters for release, ensuring specificity with sensor kinases and receptors (Table 3) (Waters and Bassler 2005).

**Table 3:** List the QS systems of common Gram‐positive pathogens and their autoinducers.

Bacteria	QS system	Signal molecule	Gene involved	Phenotype	Reference
*Bacillus anthracis*	LuxS	AI‐2	*luxS, pagR, lef, pagA, cya*	Virulence	Bozue et al. (2012)
*Bacillus cereus*	PlcR–PapR and NprR–NprX	Pentapeptide (PapR_5_), heptapeptide (PapR_7_)	*plc, lacZ*	Toxin secretion, hemolysin	Yehuda et al. (2018)
*Bacillus subtilis*	ComQXP system and Rap–Phr system	ComX, PhrC	*srfA, comS*	Surfactin production and biofilm formation	Bareia et al. (2018) and Hamoen (2003)
*Borrelia burgdorferi*	LuxS	AI‐2	*luxS, Erp*	Biofilm formation, virulence factor, and H‐binding lipoproteins production	Stevenson and Babb (2002)
*Clostridium difficile*	LuxS	AI‐2 4‐hydroxy‐5‐methyl‐3(2H) furanone (MHF)	*rolA, rolB*	Toxin production	Carter et al. (2005)
*Enterococcus faecalis*	Fsr system and LuxS system	AI‐2 Gelatinase biosynthesis activating pheromone (GBAP)	*ef1097, ef1097b, fsr* operon, *gelE, sprE*	Production of gelatinase, enterocin, and serine protease	S. G. Ali, Ansari, et al. (2017)
*Listeria monocytogenes*	LuxS, Pfs	AI‐2	*agrBDCA*	Biofilm formation	Garmyn et al. (2009)
*Staphylococcus aureus*	Agr system	AIP	*garA, agrB, agrC, agrD, nuc*	Hemolysins, phenol‐soluble modulins, adherence, and aggregation	Butrico and Cassat (2020)
*Staphylococcus epidermidis*	Agr system	AIP	*atlA, atlE, sarX*	Coding for proteases, haemolysins, and toxins	Rowe et al. (2011)
*Streptococcus mutans*	Com system	SigX‐inducing peptide (XIP)	*cin‐box*	Competence and virulence	Shanker and Federle (2017)
*Streptococcus pneumoniae*	ComABCDE	Competence‐stimulating peptide (CSP)	*comX*	Biofilm formation and production of bacteriocins	Shanker and Federle (2017)

Abbreviations: AI‐2, autoinducer‐2; AIPs, self‐inducing peptides; QS, quorum sensing.

**Figure 4:**
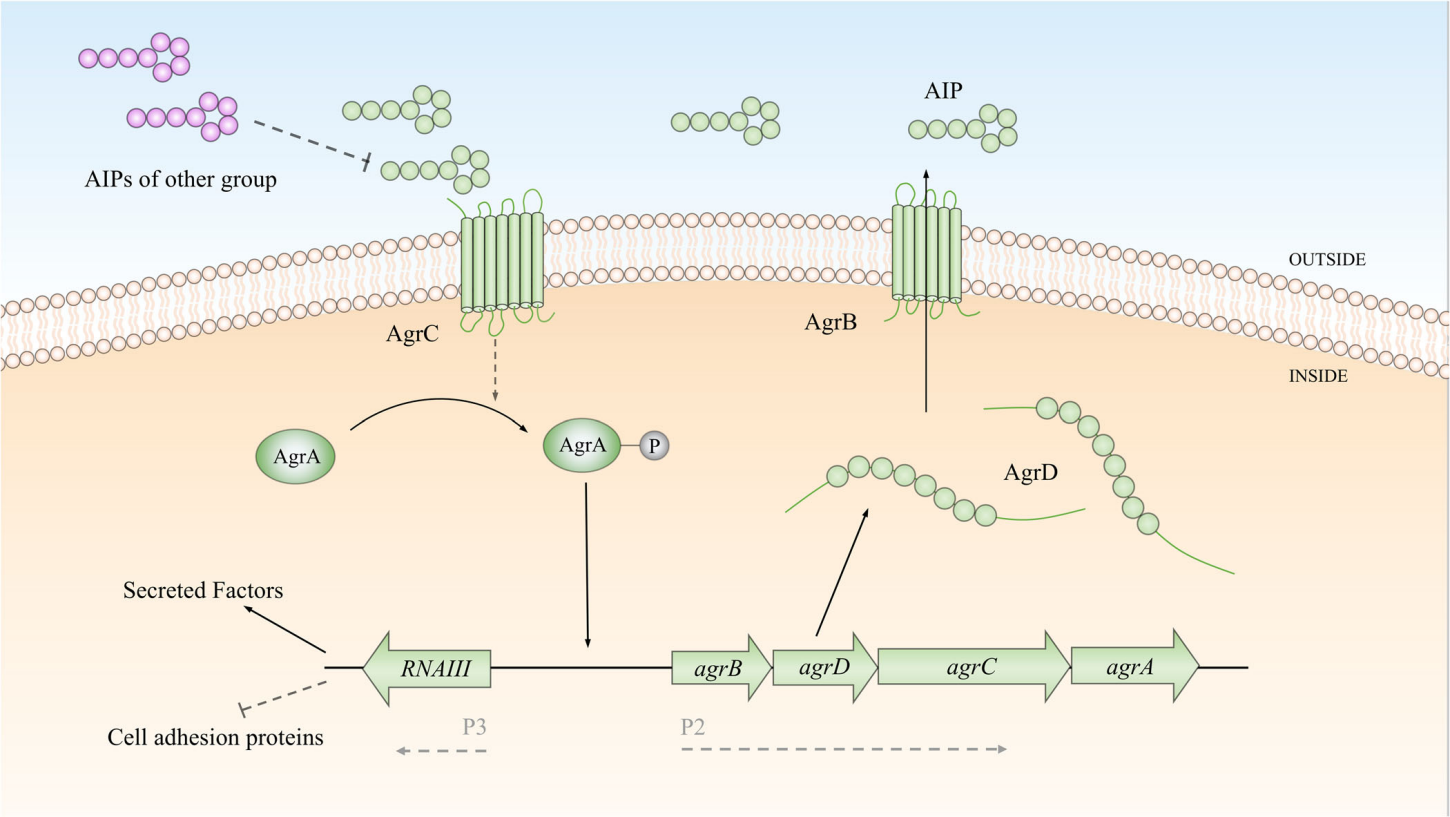
Two‐component sensor system for *Staphylococcus aureus*. P2 and P3, respectively, represent the promoters of operon *agrBDCA* and RNAIII transcription. Small circles with lactone ring structure indicate AIP produced by AgrB processing AgrD propeptide, and different colors represent AIP of different *Staphylococcal* strains origin. AIPs, self‐inducing peptides.

Among Gram‐positive bacteria, *Staphylococcus aureus* is most closely associated with human diseases. *S. aureus* causes disease by producing toxins and enzymes that damage tissues and lead to infections, especially in people with weakened immune systems (Lowy 1998; Turner et al. 2019). The Agr system, part of 16 two‐component systems in *S. aureus* (Bleul et al. 2021), is central to the regulation of virulence (Matsumoto et al. 2021) (Figure 4). The Agr two‐component system in *S. aureus* consists of transcription units driven by two promoters, P2 and P3. The system functions by producing a signal peptide, AIP, which is modified and exported outside the cell. The approximately 45‐peptide remnant AgrD is processed, cyclized, and exported as AIP by the transmembrane endopeptidase AgrB. Once AIP reaches a threshold level, it binds to the histidine kinase AgrC, phosphorylating AgrA, which in turn regulates the P2 and P3 promoters to induce gene expression. This triggers a cascade that increases AIP levels, ensuring that an HCD is maintained (Reynolds and Wigneshweraraj 2011). RNAIII facilitates the production of the toxin α‐hemolysin. During the activation of the *agr* operon, adhesin molecules are downregulated, while biofilm‐degrading enzymes are upregulated. These shifts in gene expression enable *S. aureus* to transition from an adherent state to dispersion and proceed to its virulence phase (Dastgheyb et al. 2015; Novick et al. 1993). In addition to the Agr system, other two‐component systems such as Sae, Sar, and Srr can influence *S. aureus* virulence through either affecting the Agr system or directly regulating the expression of virulence factors. As a result, the Agr system is subject to modulation by various environmental factors (Morrison 2012; Pragman et al. 2004; Voyich et al. 2009). Changes in the local environment during *S. aureus* infection can influence its pathogenicity (Mayville et al. 1999; Novick 2003). Therefore, when regulating virulence through QS, it is essential to account for the complexity of environmental variations.

Research has revealed the complexity of Gram‐positive QS systems. The infection caused by *S. pneumoniae*, the leading source of community‐acquired pneumonia, is closely linked to QS. Among the most extensively studied systems is the ComABCDE system, which resembles the Agr system of *S. aureus* (Table 3) (Karlsson et al. 2007). This system facilitates biofilm formation and bacteriocin production (Shanker and Federle 2017). In addition, the Com system is associated with horizontal gene transfer, a critical factor in *S. pneumoniae* pathogenesis and antibiotic resistance (Marks et al. 2012). Research by Domenech et al. explored disrupting the Com system to mitigate *S. pneumoniae* resistance (Domenech et al. 2020). Beyond the Com system, *S. pneumoniae* also possesses the BlpABCSRH and LuxS/AI‐2 systems. The BlpABCSRH system regulates the production of class II bacteriocins and immunity proteins (Knutsen et al. 2004; Reichmann and Hakenbeck 2000), while the LuxS/AI‐2 system influences biofilm formation on abiotic surfaces and is linked to upper respiratory tract infections (Vidal et al. 2011). Studies suggest that all three systems are involved in fratricide, a behavior that enables *S. pneumoniae* to suppress competing bacteria (Dawid et al. 2007; Steinmoen et al. 2002). Furthermore, increasing evidence indicates that the LuxS/AI‐2 system is not exclusive to *S. pneumoniae* but is also widely present in other Gram‐positive bacteria, such as *S. aureus* (D. Yu et al. 2012), *Bacillus anthracis* (Bozue et al. 2012), *Borrelia burgdorferi* (Stevenson and Babb 2002), *Clostridium difficile* (Carter et al. 2005), and *Enterococcus faecalis* (L. Ali, Goraya, et al. 2017) (Table 3). The AI‐2 molecule significantly influences biofilm formation (Vinodhini and Kavitha 2024), cell adhesion, virulence, and antibiotic resistance (Y. Wang et al. 2019) in Gram‐positive bacteria. Because AI‐2 serves as an interspecies signaling molecule, blocking these processes to influence the virulence of these pathogens may present a novel research direction. It is noteworthy that the QS systems of both Gram‐negative and Gram‐positive bacteria often regulate downstream phenotypes such as virulence and biofilms through multiple mechanisms, rather than a single circuit acting in isolation (Miyamoto et al. 2000).

## 4 Quorum Sensing Quenching and Human Disease Treatment

As the QS system serves as a crucial communication pathway for pathogenic bacteria that threaten human health, blocking QS to protect human health is therefore a vital research direction. The applications of QS include the use of biosensors for bacterial detection, the disruption of biofilm formation to prevent antibiotic resistance, and the development of potential antitumor strategies.

### 4.1 Quorum Quenching (QQ) and Quorum Sensing Inhibitors

Since the 1940s, antibiotic overuse has spurred microbial resistance to antibiotics. The intense selection pressure brought about by excessive antibiotic usage has led to the continuous emergence of drug‐resistant strains (Bell et al. 2014). Selection pressure caused by antibiotics is unavoidable, necessitating the frequent development of new antibiotics (Kalia et al. 2007). QQ offers a resistance‐free solution through targeting QS systems to reduce bacterial pathogenicity (Paluch et al. 2020).

**Figure 5:**
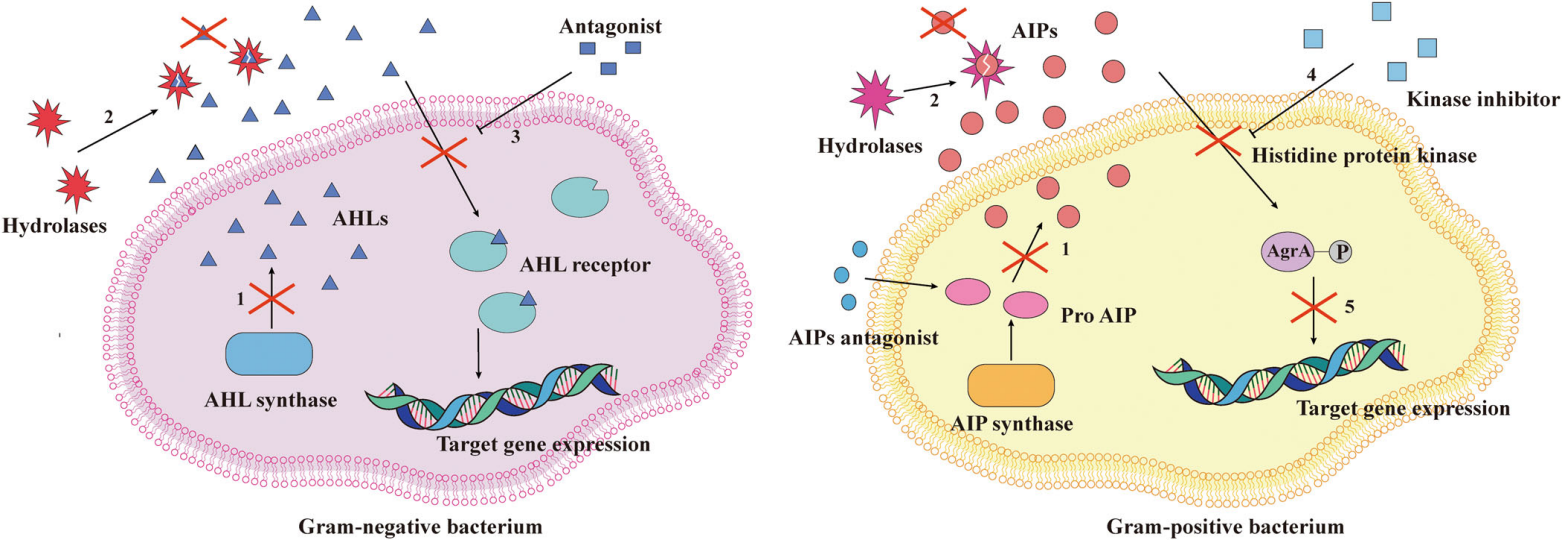
The mechanism of the three QSI. On the left are Gram‐negative bacteria. Triangles represent AHL signaling molecules. Rectangles represent receptor antagonists. Ellipses represent proteins. On the right are Gram‐positive bacteria. Red circles represent AIP, and blue circles represent AIP antagonists. Rectangles represent kinase inhibitors. The pink ellipse represents the propeptide, and the purple ellipse represents the protein. (i) Targeting signaling molecules. (1) Inhibits the synthesis of signaling molecules. (2) Degradation of signaling molecules to prevent aggregation. (ii) Targeting signaling molecule receptors. (3) Competitively binding receptors. (iii) Interference with downstream signaling cascades. (4) Prevents kinase sensor activation. (5) Blocking the binding of AgrA to DNA. AHL, acyl‐homoserine lactone; AIPs, self‐inducing peptides; QSI, quorum sensing inhibitor.

Generally, QS inhibitors (QSIs) lead to QQ through three main mechanisms. The first is targeting signaling molecules (Anandan and Vittal 2019; Chung et al. 2011). This mechanism includes the inhibition of the production of signaling molecules (Hirakawa and Tomita 2013) and the degradation of signaling molecules by QQ enzymes. Between the two, the most well‐known mechanism is the degradation of signaling molecules. Typical QQ enzymes include lactonases, acyltransferases, oxidases and reductases (Rehman and Leiknes 2018). QQ enzymes degrade signaling molecules, preventing their accumulation to threshold levels and thereby effectively blocking the onset of QS. The second mechanism targets the receptors of signaling molecules. This involves using antagonists to competitively block the binding of signaling molecules to their receptors (Bodede et al. 2018; Proctor et al. 2020). The final mechanism involves disrupting the downstream signaling cascade. This is primarily achieved through kinase inhibition (Brackman and Coenye 2015) and interference with the transcriptional regulators of operon genes (Figure 5) (Sully et al. 2014).

The applications of QQ are diverse and promising. *V. harveyi*, a major marine pathogen, threatens shrimp and causes economic losses in aquaculture (Sheikh et al. 2022). The rise of antibiotic resistance underscores the potential of targeting the QS system to combat this issue. Research has shown that AI‐2 signaling molecules in *V. harveyi*'s QS system play a crucial role in regulating virulence in brine shrimp (Defoirdt et al. 2005). Inactivating LuxS or LuxP disrupts shrimp mortality (Defoirdt et al. 2008). Furanones such as (5Z)‐4‐bromo‐5‐(bromomethylene)‐3‐butyl‐2(5H)‐furanone can block LuxR (Defoirdt et al. 2007). This way of interfering with the QS circuit does not increase the selective pressure to keep more resistant strains alive as with antibiotics, and thus can reduce pathogenicity without developing resistance (J. Chen et al. 2020). In addition, QQ can inhibit the luminescence of *V. harveyi* and the expression of toxin T1, thereby reducing the infectivity of *V. harveyi* to shrimp and its impact on aquaculture (Manefield et al. 2000). Reducing the AHL signal concentration using the AiiA enzyme can inhibit the virulence of *Erwinia carotovora* and thus improve the soft rot resistance of common vegetables, such as cabbage and potato (Dong et al. 2000). The discovery of farnesol in *Candida albicans* has extended the QS range to fungi (Hornby et al. 2001). Increasing the concentration of farnesol can inhibit the formation of *Candida* biofilm and decrease the resistance (Zibafar et al. 2009). Considering the drug resistance issues associated with antibiotic misuse, the innovative mechanism of QQ holds broader potential, making it an exciting and promising research avenue (Table 4). However, clinical QQ application demands rigorous validation due to potential toxicity and resistance risks (Bjarnsholt and Givskov 2007; Maeda et al. 2012), necessitating thorough safety assessments and strategies to address ecological and practical challenges (Maddela et al. 2019). Furthermore, research suggests that employing QQ to treat bacterial infections may lead to issues similar to antibiotic resistance (Defoirdt 2018), which contrasts with the ideal scenario outlined above. Given these limitations, future research on QQ and QSI must aim for a deeper understanding and resolution of these issues.

**Table 4:** Advances in research to suppress pathogenicity by blocking quorum sensing.

Bacteria	Inhibitor	Function	Reference
Inhibit the synthesis of AHLs			
*Pseudomonas aeruginosa*	Enoyl‐acyl carrier protein reductase inhibitors	Inhibition of enoyl‐acyl carrier protein reductase in the bacterial type II fatty acid synthesis pathway	Yang et al. (2010)
*P. aeruginosa* PA14	(2‐Nitrophenyl) methanol derivatives	Reduction the production and pathogenicity of pyocin in pathogenic bacteria	Storz et al. (2014)
*Streptococcus suis* serotype 2	Peptide TNRHNPHHLHHV	Inhibition of LuxS	Han and Lu (2009)
Hydrolyzing or modifying QS signaling molecules with QQ enzymes.			
*Bacillus velezensis* DH82	Lactonase YtnP	Degrades *N*‐acyl homoserine lactones, significantly inhibits EPS formation, biofilm formation, and production of virulence factors by *P. aeruginosa*	Sun, Hill et al. (2021)
*Bacillus* sp. AA1EC1	a. Lactonase YtnP b. Metallo‐beta‐lactamase c. Penicillin acylase	Attenuate the QS‐mediated virulence factors of agriculture relevant phytopathogens through the enzymatic degradation of AHLs	Roca et al. (2024)
*Psychrobacter* sp. M9‐54‐1	Acylase Ahap	Degrade substituted and unsubstituted AHLs from C_4_‐ to C_14_‐HSL	Reina et al. (2021)
*Psychrobacter* sp. MSB‐28	Lactonase	Inhibit the QS mechanism and biofilm formation of diverse bacterial pathogens	Packiavathy et al. (2021)
*Rhodococcus erythropolis* W2	Lactonase QsdA	Degrade *N*‐acyl homoserine lactone	Uroz et al. (2008)
*Ralstonia* sp.	Lactonase AiiD	Hydrolyze the AHL amide, releasing homoserine lactone and the corresponding fatty acid	Lin et al. (2003)
*Tenacibaculum* sp. 20 J	Lactonase Aii20J	Reduce oral biofilm grown in vitro from saliva and in a mixed biofilm of six oral pathogens	Mayer et al. (2015) and Parga et al. (2023)
Unknown	Oxidoreductase BpiB09	Reduce the 3‐oxo‐C_12_‐HSL molecules; interferes with the synthesis of the autoinducers itself by reducing the free 3‐oxo‐ acyl carrier protein in the cell	Bijtenhoorn et al. (2011)

Abbreviations: AHL, acyl‐homoserine lactone; HSL, homoserine lactone; QQ, quorum quenching; QS, quorum sensing.

### 4.2 Biosensor and Human Disease Prevention

QS‐based biosensors can rapidly and accurately assess bacterial populations in diverse settings, including clinical, food production, and environmental monitoring contexts. By detecting QS molecules, these biosensors enable the early detection of bacterial contamination, facilitating timely interventions to prevent infections and spoilage (C. Miller and Gilmore 2020; Paluch et al. 2020).

Specifically, biosensors need to be highly sensitive to HSL signals in the QS circuits of pathogenic bacteria, detecting specific pathogens via homologous binding proteins, such as LuxR. Water quality is crucial for human health, making biosensors valuable for the efficient detection of waterborne pathogens. Researchers have developed sensor modules, such as QscR, for detecting bacterial contamination in water (Wu et al. 2021). In the field of food safety, biosensors can be employed to monitor food decay based on luminescence responses to volatile organic compounds, although further development is needed to enhance the QS signal response (Veltman et al. 2022). The *V. harveyi*‐based whole‐cell biosensor can detect the AI‐2 signaling molecule expressed by *Campylobacter jejuni*, which reduces the detection limit by 100 times and can be employed for quantitative detection and the improvement of food production safety (Ramić et al. 2022). Whether the application of QS circuits or homologous binding proteins can be influenced to improve the universality in future studies is a key question. The application of QS sensors can be widely utilized for bacterial detection in food and clinical settings, with profound implications for the prevention and treatment of human diseases (Fan et al. 2022).

### 4.3 Biofilm and Resistance

Because traditional antimicrobial drugs directly target bacteria, bacterial resistance is easily induced in environments with high selection pressure (Jantaruk et al. 2021). QS‐based strategies disrupt communication, reducing virulence without killing bacteria, thereby potentially enhancing antibiotic efficacy and offering a promising route for next‐generation treatments (Paluch et al. 2020; X. Yu et al. 2021). In addition, the ability to modulate QS signaling could potentially enhance the effectiveness of existing antibiotics, making them more potent against antibiotic‐resistant bacterial strains (Rogers et al. 2012). Thus, leveraging QS in biosensors and therapeutic interventions presents a multifaceted approach to combat bacterial resistance and improve public health outcomes.

Biofilms are complex and highly structured communities of microorganisms that attach to surfaces (Costerton et al. 1995). Biofilms can grow on a variety of surfaces, both living and nonliving, such as tissues and organs, medical devices, teeth, and rocks (Li and Zhao 2020). Biofilms provide numerous advantages to microorganisms, including protection from environmental stressors (e.g., antibiotics and immune responses), enhanced nutrient availability, and a platform for cooperative behaviors (Miquel et al. 2016). Biofilm formation is a gradual process that involves successive stages, namely, bacterial adhesion to the host surface, biofilm accumulation, maturation, and eventual detachment (Stoodley et al. 2002).

When bacteria encounter a suitable surface for survival, they can attach to a surface to form a biofilm. Bacterial biofilms are extremely important (Kolter and Greenberg 2006), being closely related to pathogenicity (Woelber et al. 2022), resistance (C. W. Hall and Mah 2017), and biotechnology (Edel et al. 2019). Studies have shown that more than 80% of human pathogen infections are associated with biofilm formation (Bryers 2008). Bacteria transmit attachment signals through QS. Different bacteria exhibit variations in biofilm formation. For example, *P. aeruginosa* forms biofilms at an HCD, whereas *Vibrio cholerae* and *S. aureus* form biofilms at an LCD (Bronesky et al. 2016; de Kievit and Iglewski 2000; Waters et al. 2008). QS plays an important role in the biofilm formation of both Gram‐positive and Gram‐negative bacteria. The expression of genes responsible for biofilm formation can be controlled by regulating bacterial QS (Khan et al. 2019). Once these QS circuits are affected, bacterial colonization, adhesion, and the production of virulence factors are greatly reduced (Y. Zhang et al. 2018). Biofilms provide pathogens with a physical barrier that can resist the host's immune system and external adverse factors. Therefore, biofilm formation is crucial for the development of drug resistance. Using small molecules to block the QS circuit to affect biofilm formation is considered a potential strategy for reducing bacterial pathogenicity.

The QS system in *P. aeruginosa*, particularly the Las and Rhl system, plays a crucial role in regulating biofilm formation through influencing virulence factors, such as rhamnolipids (Smit 2003). Research indicates that rhamnolipids controlled by the Rhl system can impact biofilm aggregation (Tielker et al. 2005; Winzer et al. 2000), with mutant strains exhibiting different biofilm structures compared with wild‐type strains (Passos da Silva et al. 2017). Various factors such as AHL analogs (Hentzer et al. 2002), lectins such as LecA and LecB (Tielker et al. 2005; Winzer et al. 2000), and iron siderophores (Banin et al. 2005) also play roles in biofilm formation. The PQS system increases extracellular DNA content, interacting with extracellular polymeric substances to form biofilms (Allesen‐Holm et al. 2006; Diggle et al. 2003; Yang et al. 2007). In addition, the *luxS* gene has been linked to biofilm formation in various bacteria, including *S. aureus* (Xue et al. 2013), *Helicobacter pylori* (Cole et al. 2004), and *Streptococcus mutans* (Merritt et al. 2003), in which it has inhibitory effects on biofilm formation.

In summary, QS regulates bacterial detachment from mature biofilms, enabling their dispersion to new sites for colonization and biofilm formation. This QS‐biofilm interplay is a compelling area of research, highlighting bacterial communicate, and environmental adaptation. Reducing biofilm formation is crucial for developing bacteria‐resistant medical devices (Paluch et al. 2018) and antisepsis and anti‐biofilm metals (Piecuch et al. 2016), as well as for disrupting bacterial virulence, with broad implications for combating biofilm‐related infections and controlling bacterial growth in industrial systems.

### 4.4 Cancer

Cancer is a critical noncommunicable disease (Bray et al. 2018) that necessitates the development of innovative therapies due to treatment resistance. The use of bacterial‐mediated cancer therapy and QS to develop tumor‐targeted drug‐killing tools can minimize side effects (Patel and Spassieva 2018). Studies have shown that the specific microenvironment in necrotic tissues such as tumors can attract the preferential aggregation of *Salmonella* (Forbes et al. 2003). By utilizing this characteristic, the selective expression of therapeutic proteins activated by QS after the accumulation of *Salmonella* in tumors can minimize damage to healthy tissues (M. Zhang et al. 2014). This approach overcomes the limitations of conventional therapies and does not rely on tumor surface markers. *Salmonella* at threshold concentrations in tumors triggers QS gene expression, selectively releasing therapeutic proteins within the cancer environment (Pawelek et al. 2003). Aganja et al. enhanced *Salmonella*'s anticancer potency by detoxifying, attenuating virulence, and utilizing QS for tumor‐targeted toxin expression, thus avoiding damage to healthy tissues (Aganja et al. 2024). The specific expression range and limited triggering factors allow *Salmonella* to minimize side effects (Ganai et al. 2009; Loessner et al. 2007).

In addition to *Salmonella*, many additional bacteria, such as *Cyanobacteria* (Liang et al. 2021) and *Enterococcus faecium* (Wynendaele et al. 2022), have also been found to hold promise for cancer therapy. In conclusion, the specific expression of drug proteins by QS can avoid side effects while exerting cancer control. However, several challenges remain, including preventing the spread of bacterial infections after antitumor activity (Forbes et al. 2018), achieving the highest possible bacterial targeting to improve specificity, and managing various virulence factors induced by bacterial QS or immune responses triggered by the bacteria itself (Kalia et al. 2022). As a result, most studies utilize attenuated or detoxified strains that have been screened to ensure the health of the organism (Kalia et al. 2022; Kumar et al. 2022).

Today, humanity faces the critical challenge of rising antibiotic ineffectiveness (Kwon and Powderly 2021). However, research on QQ enzymes and QSI offers new hope for overcoming this predicament. Blocking biofilm formation and expression of virulence factors by interfering with the QS system may avoid resistance resulting from the survival of resistant strains under selective pressure. This nonselective pressure strategy holds promise as a potential antibiotic alternative (Kalia et al. 2019) and offers a new avenue for addressing the challenges of treating cancer (Liang et al. 2021). Whether in clinical applications (Borges and Simões 2019; Carradori et al. 2020) or industrial settings (Defoirdt et al. 2011; Skindersoe et al. 2008), QS regulatory technologies have demonstrated significant potential. However, compared with controlled laboratory conditions, the bacterial communities and QS networks in real‐world environments are far more complex. Further research is essential to enhance the specificity and targeting accuracy of intricate QS systems, paving the way for more effective and safe applications.

## 5 Novel QS Regulation Mode

The greater the harm that bacteria pose to human health, the more significant QS becomes in disease prevention and control. Currently, many novel and unexpected QS regulatory mechanisms have been discovered. For example, new materials such as nanomaterials and hydrogel materials can be used in combination with QS. In addition, to protect the environment and human health through QS degradation of microplastics (MPs) by bacteria. Furthermore, the use of phages to protect humans is also a promising direction. These new solutions will greatly improve the application scope and application value of QS.

### 5.1 Nanomaterial Regulation

We are currently in the postantibiotic era. QS techniques hold promise in mitigating bacterial virulence without triggering resistance, making research on QSI and QQ enzymes highly active. However, recent concerns have been raised regarding the low bioavailability and high toxicity of QSI (Gómez‐Gómez et al. 2019). Nanomaterials, with their superior properties, not only have the potential to directly modulate QS but can also significantly enhance the efficiency and stability of QSI. Therefore, nanocomposites make QQ more feasible.

Nanomaterials are widely utilized in various applications. Depending on their distinct properties, nanomaterials can serve as optical sensors (Vasudevan et al. 2021), electrochemical sensors (Capatina et al. 2022), and bacterial biosensors (Guan et al. 2018), enabling the detection and diagnosis of diseases when bacterial populations reach a certain threshold. Nanomaterials can prevent bacterial colonization and spread (Roy et al. 2022) and inhibit the secretion of virulence factors (Lu et al. 2020). In addition, nanomaterials can disrupt and inhibit biofilm formation. This results in a significant reduction in bacterial virulence and defense mechanisms, making prevention and treatment more straightforward and effective (Kamli et al. 2021).

**Figure 6:**
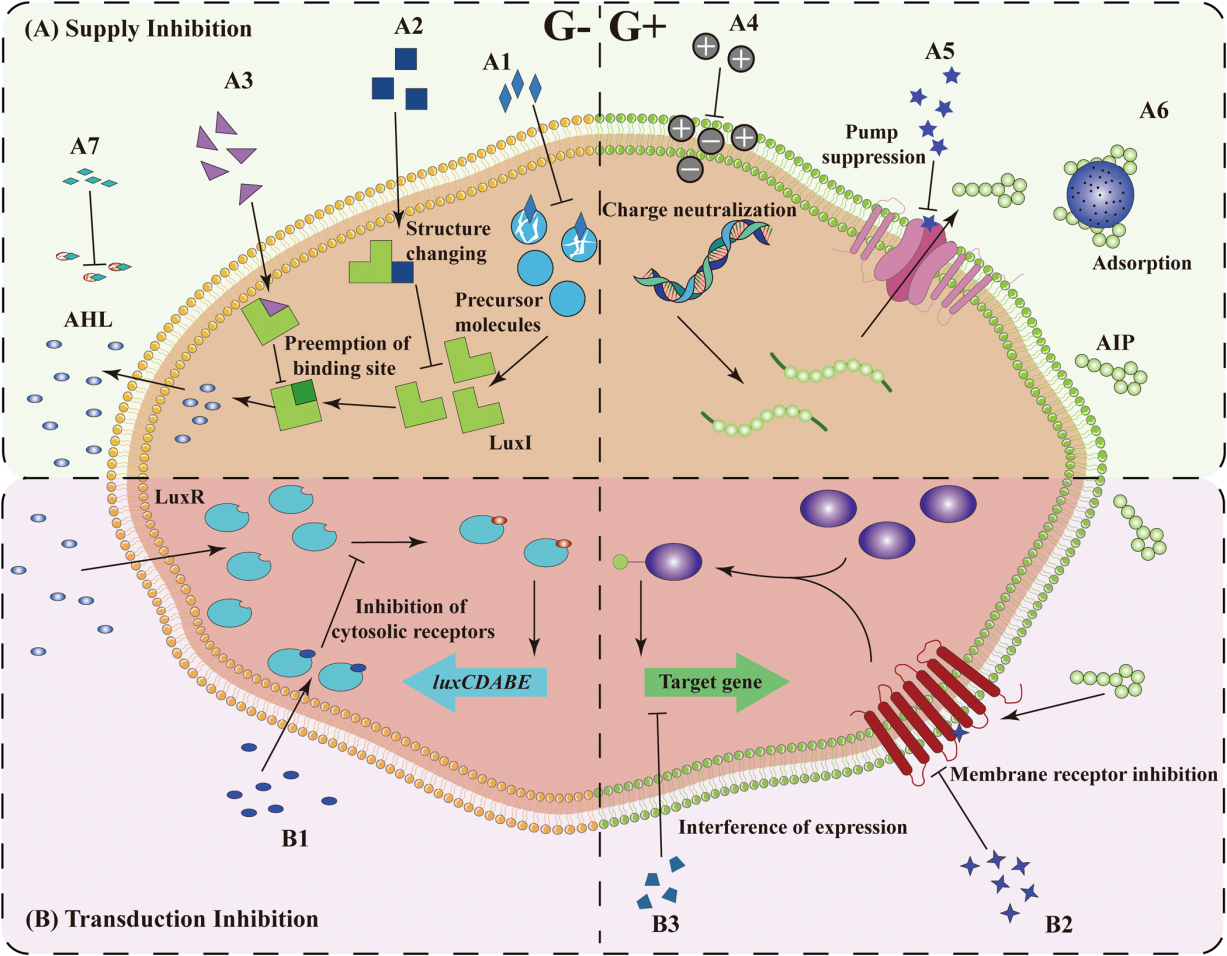
Mechanisms of nanomaterial regulation in QS. The green box (A1–A7) represents signal supply inhibition, while the purple box (B1–B3) represents signal transduction inhibition. The left side illustrates Gram‐negative bacteria, and the right side illustrates Gram‐positive bacteria. See the text for detailed mechanisms. AHL, acyl‐homoserine lactone; AIPs, self‐inducing peptides; QS, quorum sensing.

Nanomaterials primarily interfere with QS through inhibiting the supply and transduction of signaling molecules (Figure 6). In terms of the signal supply, nanomaterials can block the supply of precursor molecules (A1) (Shah et al. 2019), alter the conformation of enzymes such as LuxI synthase (A2) (S. G. Ali et al. 2017), or block the active sites of these enzymes (A3) (Shah et al. 2019). Nanomaterials also prevent the secretion of signaling molecules into the extracellular space via inducing aggregation effects (A4) (Omwenga et al. 2018) and suppressing efflux pumps (A5) (Ahmed et al. 2021). In addition, nanomaterials can adsorb signaling molecules due to their adsorptive properties (A6) (Piletska et al. 2010) or catalyze the degradation of signaling molecules (A7) (Jegel et al. 2021). This prevents the extracellular accumulation of QS signals, hindering their ability to reach the threshold necessary to trigger the expression of virulence factors. In terms of signal transduction, nanomaterials can competitively bind to cytoplasmic and membrane receptors (B1, B2) (Balakrishnan et al. 2020; Omwenga et al. 2018), disrupting the functionality of the cell membrane. In addition, nanomaterials can even directly damage DNA to inhibit gene expression (B3) (L. Wang et al. 2017).

However, due to constraints in nanomaterial processing and delivery methods, the current application of nanomaterials to regulate QS is primarily limited to treating diseases of the digestive and respiratory systems (Mogosanu et al. 2015). Expanding nanomaterial modification techniques could greatly enhance their therapeutic value. For example, injectable nanomaterials could be utilized to treat bloodborne or systemic bacterial infections (Peng et al. 2024), or topical applications could be absorbed through the skin to treat bone or joint disorders (Cheng et al. 2022). This expansion would require focused research on nanomaterial dosage and safety. In addition, nanomaterials currently face issues with stability, which presents challenges in storage and transportation. Most importantly, the microbial environment in the human body is far more complex than that in a laboratory setting, necessitating extensive experimentation to validate the effectiveness of nanomaterials.

### 5.2 Hydrogel Regulation

Hydrogels are an intriguing material that has garnered increasing research interest in recent years. Currently, the design of hydrogels allows them to respond to a variety of environmental changes. For example, hydrogels can release specific substances in response to stimuli, such as temperature, pH, or light, thereby regulating or inhibiting bacterial QS. This represents a novel approach for treating many infectious diseases.

Research on utilizing hydrogels to regulate QS across various dimensions has highlighted its advantages, such as the inhibition of biofilm formation (Hu et al. 2022; Khaleghi et al. 2023), the suppression of virulence factor production (Prateeksha et al. 2019), anti‐inflammatory properties (Tu et al. 2022), anti‐infection efficacy (Qu et al. 2024), detection capabilities (Seto 2019), and applications in food packaging and seafood preservation (D. Wang et al. 2023). For example, high concentrations of reactive oxygen species (ROS) produced by bacterial infections can exacerbate inflammation, thereby hindering the treatment of posttraumatic osteomyelitis (W. Zhang et al. 2024). Zhang et al. synthesized a hydrogel that releases hyperbranched polylysines (HBPLs) in response to changes in ROS and pH levels in the environment. Under acidic conditions and in the presence of high H_2_O_2_ concentrations, the hydrogel releases HBPL more rapidly. HBPL effectively scavenges ROS, disrupts bacterial biofilm formation, and interferes with the expression of QS‐related genes (W. Zhang et al. 2024). In addition, Seto developed a hydrogel capable of detecting *P. aeruginosa* infections in a cell‐free environment. This cell‐free compatible hydrogel demonstrates very high sensitivity and practicality (Seto 2019).

Due to their unique viscoelasticity and rheological properties, hydrogels are widely employed in the field of delivery systems. This suggests that incorporating stimuli‐responsive agents and therapeutic substances such as QSI into hydrogels could enable the disruption of QS, thereby achieving therapeutic outcomes. However, research on the use of hydrogels for QS regulation remains limited, and whether hydrogels can realistically serve as an alternative to antibiotics, offering a novel therapeutic approach, requires extensive experimental validation in further research.

### 5.3 QS and MP Degradation

MPs are defined as plastic particles or fragments with a diameter of less than 5 mm (Thompson et al. 2004). Today, MPs are distributed globally (Ivar do Sul et al. 2009) and pose serious risks to organisms within the environment (Fueser et al. 2020). As apex predators in the food chain, humans can accumulate significant amounts of MPs in their bodies through biomagnification, leading to potential harm (Barboza et al. 2018; Vethaak and Legler 2021). Leveraging bacterial QS holds promise for addressing this issue.

Some bacteria utilize MPs as a transport vector, attaching to the surface of MPs to form biofilms and create a plastisphere, which enables the long‐distance transport of microorganisms (Zettler et al. 2013). The plastisphere is a miniature ecosystem that can harbor a variety of microorganisms (Amaral‐Zettler et al. 2020). As a result, MPs facilitate the diffusion of microorganisms and influence aquatic ecosystems (Ahmad et al. 2020). *Rhodobacteraceae* are abundant in the plastisphere, likely due to the crucial regulatory role of conserved QS signals in biofilm formation (Debroas et al. 2017; Zan et al. 2014). As mentioned earlier, targeting QS to degrade MPs may represent a key research direction. *Bacillus cereus* has been shown to play an important role in the degradation of polyethylene, polyethylene terephthalate, and polyethylene terephthalate (X. Xu et al. 2023). As demonstrated by Su et al. through experimentation, QS may be a significant factor influencing the degradation of marine particulate matter (Su et al. 2018). However, research on the QS‐mediated degradation of MPs remains limited and lacks comprehensive understanding. Because QS can enhance the bacterial recognition and degradation of MPs, introducing QS signaling molecules or analogs to activate bacterial degradation pathways represents another promising research direction. Furthermore, employing genetic engineering and other means of modifying the QS system to enable the expression of additional MP‐degrading enzymes is a promising approach. In conclusion, the use of bacterial QS systems will ameliorate the effects of MPs on the marine environment. More importantly, reducing the threat of MP has a great effect on human health.

### 5.4 Virus Monitoring

Given the increasingly serious issue of antimicrobial resistance and a growing body of research on interkingdom QS, bacteriophages have received increasing attention (Luong et al. 2020). Some phages lay dormant until activated by signals from the bacterial communication system. Virulent phages (Suramo et al. 1981) replicate and destroy bacteria, while temperate phages (Hay and Lithgow 2019) can either kill the host (Ofir and Sorek 2018) or coexist through lysogeny (Lwoff 1953). Polylysogeny allows multiple prophages in a bacterium, but DNA damage can disrupt this balance (Campbell et al. 2020). Interestingly, DNA damage does not always induce phage replication. The host response to infection and QS signals can trigger the transition from lysogenic to lytic cycles in temperate phages (Silpe et al. 2023). Phages can also “monitor” bacterial signals to determine the best time to replicate. Elucidating this process can potentially enable the use of QS regulation to induce bacteriophages to eliminate harmful bacteria, presenting a novel approach to combat infections. This approach can avoid the selective pressure caused by drugs, which gives rise to bacterial resistance. Frequent antibiotic changes are costly. Therefore, the use of bacteriophages is expected to replace antibiotics to change this situation (Jin et al. 2023). It is noteworthy that phages can monitor specific autoinducers expressed by bacteria while also being triggered for dissemination (Silpe and Bassler 2019). This means that improper control could lead to phage infection following bacterial lysis. Therefore, the application of phages requires extensive research to identify a balanced state among bacteria, viruses, and the human body. This is essential to ensure that harmful bacteria can be monitored and eliminated without compromising health.

## 6 Conclusion and Perspective

QS is pivotal in bacterial communication, with progress in gene expression and regulatory mechanisms. It facilitates microbial interactions and adaptation across ecosystems, prompting further research into its cross‐kingdom impacts. QS emerges as a strategic solution to pressing challenges, including microbial pathogenicity, antibiotic resistance, and cancer. It has the potential to mitigate pathogen virulence and regulate pathogenic bacteria through viral agents, reducing the risks of antibiotic overuse. The integration of QS with nanomaterials and hydrogels enhances its potential as a microbial control tool. However, the intricacies of QS networks and the variability in nonideal natural conditions present substantial challenges that necessitate further research and validation. In essence, QS has transcended its role as a communication mechanism to become an indispensable conduit for human modulation of microbial behavior.

## Author Contributions

**Derun Liu:** writing – original draft, investigation, conceptualization, visualization, methodology. **Yonglin Lu:** visualization, methodology. **Ziyun Li:** writing – review and editing, visualization. **Xin Pang:** visualization. **Xueyan Gao:** conceptualization, funding acquisition, writing – review and editing, project administration, supervision.

## Ethics Statement

The authors have nothing to report.

## Conflicts of Interest

The authors declare no conflicts of interest.

## Data Availability

The authors have nothing to report.
